# Mechanistic insights into the sleep-glymphopathy-cerebral small vessel disease loop: implications for epilepsy pathophysiology and therapy

**DOI:** 10.3389/fnins.2025.1546482

**Published:** 2025-03-27

**Authors:** Zaw Myo Hein, Zaid Adnan Subhi Al-Zaghal, Mazira Muhammad Ghazali, Usman Jaffer, Hafizah Abdul Hamid, Muhammad Zulfadli Mehat, Muhammad Danial Che Ramli, Che Mohd Nasril Che Mohd Nassir

**Affiliations:** ^1^Department of Basic Medical Sciences, College of Medicine, Ajman University, Ajman, United Arab Emirates; ^2^Department of Neurosciences, School of Medical Sciences, Universiti Sains Malaysia, Kubang Kerian, Kota Bharu, Kelantan, Malaysia; ^3^Kulliyyah of Islamic Revealed Knowledge and Human Sciences, International Islamic University Malaysia, Kuala Lumpur, Malaysia; ^4^Department of Human Anatomy, Faculty of Medicine and Health Sciences, Universiti Putra Malaysia, Serdang, Selangor, Malaysia; ^5^Brain and Mental Health Research Advancement and Innovation Networks (PUTRA BRAIN), Universiti Putra Malaysia, UPM Serdang, Selangor, Malaysia; ^6^Faculty of Health and Life Sciences, Management and Science University, Shah Alam, Selangor, Malaysia; ^7^Department of Anatomy and Physiology, School of Basic Medical Sciences, Faculty of Medicine, Universiti Sultan Zainal Abidin, Kuala Terengganu, Terengganu, Malaysia

**Keywords:** sleep, glymphatic system, cerebral small vessel disease, epilepsy, therapy

## Abstract

Epilepsy is the second most common neurological disorder and affects approximately 50 million people worldwide. Despite advances in antiepileptic therapy, about 30% of patients develop refractory epilepsy. Recent studies have shown sleep, glymphatic function, cerebral small vessel disease (CSVD), and epilepsy are interrelated by sharing a multidirectional relationship in influencing their severity and progression. Sleep plays a vital role in brain homeostasis and promotes glymphatic clearance responsible for the removal of metabolic wastes and neurotoxic substances from the brain. Disrupted sleep is a common feature in epilepsy and can lead to impairment in glymphatic efficiency or glymphopathy, promoting neuroinflammation and accrual of epileptogenic factors. CSVD, occurring in up to 60% of the aging population, further exacerbates neurovascular compromise and neurodegeneration by increasing seizure susceptibility and worsening epilepsy outcomes. This narrative review aims to discuss the molecular and pathophysiological inter-relationships between these factors, providing a new framework that places glymphopathy and CSVD as contributors to epileptogenesis in conditions of sleep disruption. We propose an integrative model wherein the glymphopathy and vascular insufficiency interact in a positive feedback loop of sleep disruption and increased seizure vulnerability mediated by epileptic activity. Acknowledging these interactions has significant impacts on both research and clinical practice. Targeting sleep modulation, glymphatic function, and cerebrovascular health presents a promising avenue for therapeutic intervention. Future research should focus on developing precision medicine approaches that integrate neuro-glial-vascular mechanisms to optimize epilepsy management. Clinically, addressing sleep disturbances and CSVD in epilepsy patients may improve treatment effectiveness, reduce seizure burden, and improve overall neurological outcomes. This framework highlights the need for interdisciplinary approaches to break the vicious cycle of epilepsy, sleep disturbance, and cerebrovascular pathology, paving the way for innovative treatment paradigms.

## 1 Introduction

Epilepsy is a prevalent neurological disorder which affects up to 70 million people of most populations in the world, with a significant burden on patients and healthcare systems (World Health Organization, 2024; Thijs et al., 2019). Based on the International League Against Epilepsy (ILAE), epilepsy is characterized by recurrent two or single-unprovoked seizures and is often comorbid with a range of disorders, notably sleep disorders and neurovascular abnormalities (Peng et al., 2020; Fisher et al., 2014). Strikingly, the prevalence of sleep disturbances in epilepsy patients is deemed to range from 30 to 50%, depending on the type, severity, and status of treatment of epilepsy (Matos et al., 2022; Yazdi et al., 2013). Moreover, it is estimated that the proportion of drug-resistant epilepsy cases is approximately 30% of total epilepsy cases; thus, they cause a heavy cognitive, psychological, and socio-economic burden on individuals and healthcare systems (Laxer et al., 2014; Löscher and Klein, 2022). Emerging research has unveiled a complex interplay between sleep and epilepsy concerning the brain waste clearance systems, such as the glymphatic system (Peng et al., 2024), and vascular-related neurodegenerative diseases like cerebral small vessel disease (CSVD) (Stösser et al., 2019; Turon et al., 2021). This interrelationship points out a complex pathophysiology in which disturbances in one domain may promote the others, leading to a vicious cycle able to worsen seizure frequency and neurodegeneration.

There is an increasing awareness that sleep is important both in the management of epilepsy and in the course of the underlying disease. Slow-wave sleep (SWS) was noted to have inhibitive impacts on epileptic activity. In contrast, poor sleep architecture, including decreased SWS and increased wake after sleep onset, is associated with worsened seizure control and cognitive decline in patients with epilepsy (Sheybani et al., 2023). In addition, sleep disturbances are common among epilepsy patients, whereby the prevalence was estimated to lie between 30 and 45%, depending on the type and severity of epilepsy (Thomas et al., 2010; Yazdi et al., 2013). Moreover, studies have reported that interictal epileptiform discharges (IED), which can occur during non-rapid eye movement (NREM) sleep, particularly in SWS, could contribute to the impairment in memory consolidation and increased seizure susceptibility (Sheybani et al., 2023; Fouad et al., 2022). These findings were illustrative of the need to comprehensively explore how disrupted sleep and epilepsy influence one another, especially via considerations of the glymphatic system and CSVD.

The glymphatic system is a recently characterized cerebrospinal fluid (CSF)-interstitial fluid (ISF)-based waste clearance pathway; it plays an important role in the removal of metabolic waste products in the brain via sleeping (Iliff et al., 2012). Given that this system is particularly active during SWS, disrupted sleep in epilepsy may further lead to a disturbance in glymphatic clearance, so-called glymphopathy, resulting in the accumulation of neurotoxic substances such as amyloid-beta (Aβ) and tau, which are implicated in neuroinflammation and cognitive impairment (Nedergaard and Goldman, 2020; Voumvourakis et al., 2023, p. 14). Evidence from recent studies highlighted that epilepsy-related sleep disturbances, particularly those affecting deep sleep, may compromise glymphatic function, promoting a pro-epileptic and neurodegenerative environment (Lee et al., 2022a; Lee et al., 2022b). The possible link between glymphopathy and epilepsy stirred interest in investigating whether enhancing glymphatic function can be a new therapeutic target in the management of epilepsy.

Compounding these interactions, CSVD is an age-related condition, prevalent in the small vessels of the brain, increasingly recognized as a contributor to neurodegenerative processes. CSVD, though present in up to 60% of older adults, is found to be a major risk factor for stroke, cognitive decline, and dementia and has been implicated in epilepsy development with reference to late-onset epilepsy (Turon et al., 2021; Wardlaw et al., 2019). In epilepsy patients, CSVD may enhance seizure susceptibility through mechanisms involving chronic hypoperfusion, disruption of the blood brain barrier (BBB), and inflammation (Wang et al., 2024). Impaired vascular function in CSVD might further exacerbate glymphopathy or vice versa, enhancing the neuroinflammatory processes and further increasing the severity of epileptogenesis and cognitive decline in comorbid epilepsy and CSVD patients.

This narrative review aims to critically analyze the molecular and pathophysiological mechanisms underlying the interaction among sleep, the glymphatic system, CSVD, and epilepsy. Particular attention is given to how these systems may interactively influence epilepsy evolution and cognitive outcomes. We integrate findings from basic sleep research, glymphatic studies, and cerebrovascular science to propose a comprehensive framework for elucidating these interconnections. This might provide not only a deeper look into the pathophysiology of epilepsy but also a gateway to new therapeutic strategies aimed at improving seizure control and cognitive health in patients with epilepsy through intervention targeting glymphatic and vascular functions. Hence, the insights presented here will underscore the urgent need for further research into these interrelated domains to optimize personalized and precision-based interventions for epilepsy management.

## 2 Sleep and epilepsy: mechanisms and reciprocal influences

**Figure 1:**
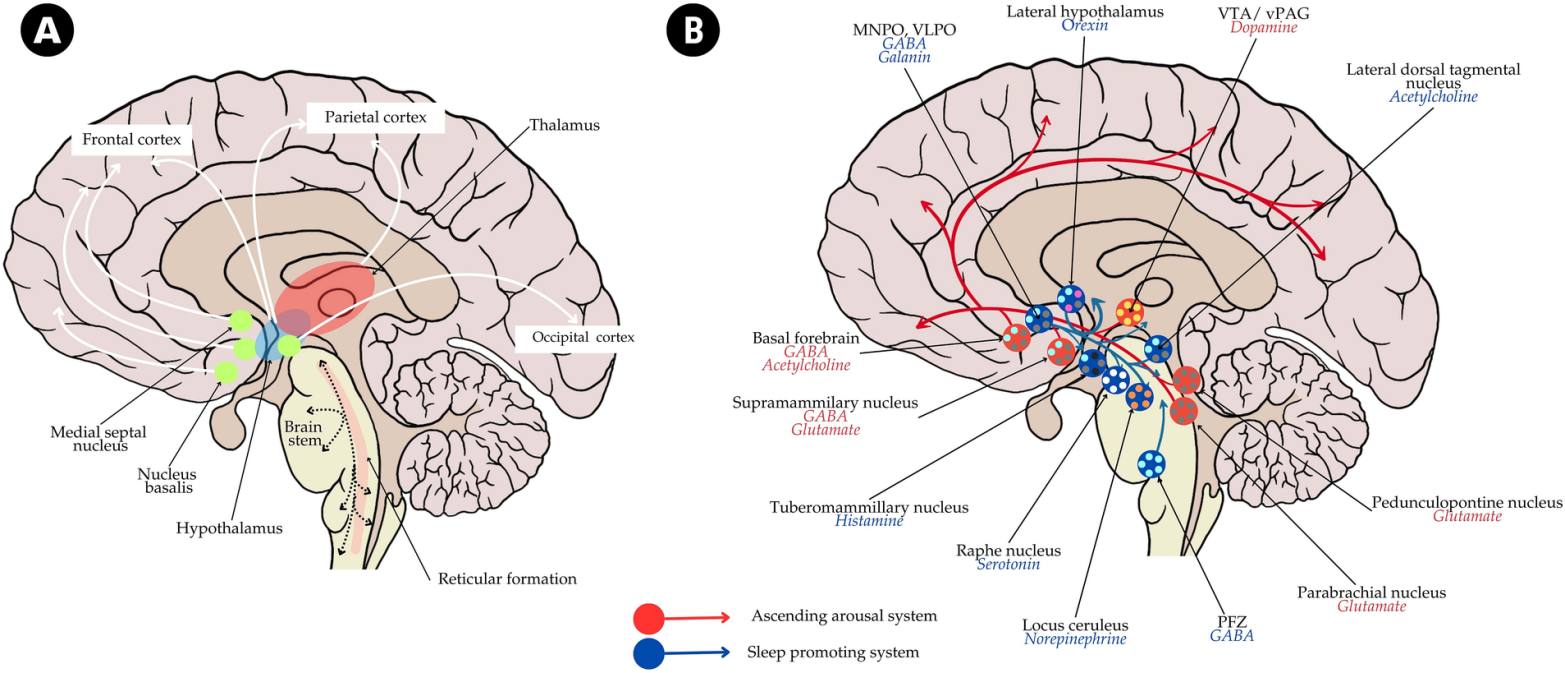
Schematic diagram of **(A)** ascending reticular activating system (ARAS) and **(B)** ascending arousal system (AAS) system with the neuronal pathway and neurotransmitter involved. The diagram also illustrates the pathway of the sleep-promoting system (in blue arrow and blue circle). MNPO, median preoptic nucleus; PFZ, parafacial zone; VLPO, ventrolateral preoptic nucleus; VTA, ventral tegmental area; vPAG, ventral periaqueductal gray.

Interactions between sleep and epilepsy reflect a complex interplay between multiple neural systems, which include the ascending reticular activating system (ARAS) and ascending arousal system (AAS) responsible for cortical activation, wakefulness, and sleep architecture (Brown et al., 2012) (see Figure 1). Epileptic activity may interfere with these sleep-arousal systems, leading to fragmented sleep, heightened seizure susceptibility, cognitive decline, and even coma (Nobili et al., 2022). In turn, distinct phases of sleep modulate the expression of epileptic activity and also neuroplasticity, thus affecting memory consolidation, mood regulation, and seizure control (Halász and Szűcs, 2020).

Interactions have been mediated through sleep-dependent molecular, genetic, and network mechanisms and are increasingly studied through advanced imaging and electrophysiological techniques, including electroencephalography (EEG), magnetoencephalography (MEG), structural and function magnetic resonance imaging (MRI) including functional MRI (fMRI), and diffusion tensor imaging (DTI). Each of these provides a different perspective on how sleep disturbances in epilepsy might interfere with neural circuits (i.e., in ARAS and AAS) and the underlying molecular mechanisms to promote epileptogenesis, thus affecting brain homeostasis.

### 2.1 Effects of sleep on epileptic activity

The interplay between the stages of sleep and the epileptic activity underlines the deep impact of the systems of arousal of the brain. During NREM sleep, especially in stages of SWS, which is characterized by high-amplitude, low-frequency synchronized oscillations across thalamocortical networks (Sheybani et al., 2023), the decreased activity of the ARAS contributes to the increased cortical synchrony in SWS, where the spread and propagation of epileptic discharges along thalamocortical networks are thus easily facilitated (Arican et al., 2021; Frauscher et al., 2015; Sheybani et al., 2023). In epilepsy, structural and functional disturbances within ARAS will lead to defects in connectivity, especially in patients subject to nocturnal seizures, thereby serving as a venue for seizure propagation during NREM sleep (Frauscher et al., 2015).

The AAS is densely interconnected with the ARAS, whereby it controls arousal and sleep transition. AAS constituted a multi-functional composition of glutamatergic, cholinergic, and gamma-aminobutyric acid (GABAergic) neurons projecting to a wide range of cortical areas. For instance, among the major excitatory components of AAS are the parabrachial and pedunculopontine tegmental nuclei; lesions in these nuclei have been linked with arousal disruption (Fuller et al., 2011; Kroeger et al., 2022). In epilepsy, disturbed glutamatergic pathways of these nuclei further destabilize arousal, increasing transitions into sleep stages that are most vulnerable to seizures (Barker-Haliski and White, 2015; Chen et al., 2023). Cholinergic and GABAergic neurons in the basal forebrain further modulate cortical arousal and sleep–wake states. Studies from animal model (rodent) shown that, abnormal activity of these neurons in epilepsy may facilitate seizure initiation and spread, especially in REM-related seizures, when typically, there is a disruption of REM patterns (Brown and McKenna, 2015; Motelow et al., 2015; Wang et al., 2021).

Seizure dynamics are differently affected by the various stages of sleep, often through variations in cortical excitability and synchronization. Genetic analyses showed that mutations in genes modulating the thalamocortical circuitries, like CACNA1H and SCN1A, coding for the calcium channel voltage-dependent T-type alpha 1H subunit protein and the sodium channel protein type 1 subunit alpha, respectively, may influence the susceptibility to NREM-related seizures, thus underlining a molecular basis for pro-epileptogenic effects of this stage of sleep (Eckle et al., 2014; Miller and Sotero de Menezes, 2022).

On the other hand, REM sleep seems to be associated with reduced seizure frequency, for which its desynchronised EEG pattern has been associated. In REM, a decrease in thalamocortical synchrony leads to reduced cortical excitability, thus, in effect, stopping the spread of epilepsy (Ng and Pavlova, 2013). MEG and fMRI studies also followed the same trend, where during REM, connections in the cortex were reduced compared to NREM (Dang-Vu et al., 2010; Ioannides et al., 2017) which might be contributing to diminished seizure activity during the sleep phase (Frauscher et al., 2015).

The sleep microstructure, such as spindles and cyclic alternating patterns (CAP), is also of critical importance in the regulation mechanisms of epileptic activity manifestations (Nobili et al., 2022). Spindles are brainwave patterns generated during light NREM sleep (N2 stage) and are related to memory consolidation due to thalamocortical coupling. Disturbances in normal spindle activity are one of the common findings among epilepsy patients; this disturbance characterizes increased IEDs indicative of heightened epileptic activity. EEG studies demonstrate that the frequency and duration of spindles are reduced in epilepsy patients. Such spindle reductions may indicate that epilepsy also disrupts the neural circuits related to spindles, leading to further impairment of sleep-dependent memory processes (Kramer et al., 2021).

Furthermore, CAP which also denotes periodic EEG activity in NREM sleep, is characterized by sequences of transient electrocortical events that differ from the tonic background and appear at regular intervals up to 1 min (Parrino et al., 2014). It is a marker of sleep instability, consisting of recurrent bursts of cortical activity alternating with lower EEG amplitude. For example, in the case of phase A1 in CAP (i.e., a specific phase characterized by high-amplitude slow waves on the EEG, signifying a period of relatively stable, deep sleep with minimal muscle activity); an increased number of epileptic spikes (demarcated by delta bursts and k-complexes, with frequency ranging from 0.5 Hz to 4 Hz) in EEG studies on epilepsy patients were present (Dhok et al., 2020). It seems that the CAP cycle itself offers optimal conditions for the development of cortical excitability of epileptic discharges during NREM sleep, especially in patients with sleep-related epilepsies. The association of CAP with IEDs illustrates how microstructural sleep disruptions can contribute to the increased risk of seizures in epilepsy.

In summary, these changes in cortical arousal due to the mutually influencing ARAS and AAS, along with states of seizure susceptibility modulated across sleep stages and microstructure, collectively point to sleep stability as a priority for epilepsy management. Structural and functional abnormalities of ARAS and AAS are commonly evidenced by neuroimaging, perturbed sleep patterns and lower seizure thresholds during sleep–wake transitions and NREM sleep. Insight into such processes therefore underlines the potential for anti-epileptic therapeutic strategies to target neurobiological sleep mechanisms, most especially by the use of techniques that are aimed at stabilizing the arousal system and the architecture of sleep.

### 2.2 Effects of epileptic activity on sleep structure

Epileptic activity, on the contrary, strongly influences sleep structure, and typically, it gives rise to very disorganized and fragmented sleep. Polysomnographic studies in patients with epilepsy have shown an increased wake-after-sleep onset (WASO) and a reduction in time spent in SWS and REM (Garg et al., 2022). EEG recorded in both human and animal models that epileptic discharges disrupted the continuity of sleep stages, which hence prevented the occurrence of deep restorative sleep stages that are important for brain homeostasis (Pearce et al., 2014; Sharma et al., 2018; Wei et al., 2021).

Neuroimaging investigations have provided further evidence in demonstrating that seizure activity disrupts normal connectivity, especially in those networks associated with sleep–wake transitions (i.e., basal forebrain and parabrachial nuclei) (Brown et al., 2012). Indeed, fMRI studies have previously demonstrated decreased AAS (i.e., thalamic and cortical) connectivity during postictal states, which could be relevant for hampered transition into deep sleep stages (McCafferty et al., 2023). This could explain the disturbed and unrefreshing sleep commonly reported by patients with epilepsy, which may further contribute to increased susceptibility to seizures and cognitive decline over time.

Added to this, there is the fact that epileptic discharges during sleep are particularly destructive to cognitive processes, like memory consolidation, dependent upon sleep-dependent mechanisms (Moore et al., 2021). Coordinating hippocampal ripples and cortical spindles with synchronized cortical slow oscillations is a SWS requirement for transferring memory traces from the hippocampus to the neocortex (Buzsáki, 2015). In contrast, IEDs during NREM disturb these oscillatory patterns and interfere with the hippocampal-neocortical dialog that forms part of long-term memory consolidation (Lambert et al., 2020). In DTI studies, patients with frequent sleep-related epileptic discharges show structural abnormalities in ARAS-related pathways (i.e., hippocampal and thalamocortical tracts) (Lin et al., 2020). These white matter disruptions correlate with memory impairment and suggest that recurrent sleep-dependent IEDs might contribute to long-term structural alterations in critical memory pathways. Electrophysiology and electrocorticography (ECoG) recordings studies also suggest that high-frequency IEDs are associated with reduced spindle density within the hippocampus, further disrupting this synchrony that is necessary for memory consolidation during sleep (Gelinas et al., 2016).

### 2.3 Circadian and homeostatic regulation in epilepsy

The sleep–wake cycle is driven by circadian rhythms governed by the suprachiasmatic nucleus (SCN), determining seizure timing. Regulated by genes such as CLOCK, BMAL1, and PER2, circadian rhythms influence neuronal excitability and, therefore, may play a critical role in modulating seizure susceptibility (Re et al., 2020). For example, mutations within the circadian genes in rodent models of epilepsy have been demonstrated to reduce seizure thresholds, thus allowing for increased seizure incidence at select times of the day (Purnell et al., 2021).

In human studies, circadian rhythms regulated seizure times, and usually can vary depending on the epilepsy type and seizures focus, i.e., which part of the brain the seizures originate from. For example, frontal lobe seizures, a type of focal seizures, occur at night, particularly during NREM sleep and temporal lobe seizures, on the other hand, tend to be more evenly distributed across the day but can still have a circadian influence depending on the patient (Jin et al., 2020). Additionally, generalized seizures affect both sides of the brain, causing widespread electrical activity, occur more in the morning (Zhong et al., 2020). Moreover, investigations into epilepsy patients showed that nocturnal seizures are associated with disrupted circadian regulations since EEG/fMRI signal changes in altered SCN-and ARAS-related connectivity affect sleep–wake stability (Liu et al., 2022). This indicates that SCN is an important circadian modulator and may point toward a neurobiological substrate underlying time-dependent patterns in epilepsy.

Moreover, sleep deprivation, which disrupts the homeostatic balance of sleep, is an established seizure precipitant in epilepsy. Studies in sleep-deprived animals demonstrate increased cortical excitability and heightened seizure susceptibility, presumably driven by changes in neurotransmitter levels, including heightened levels of glutamate and lessened activity of gamma-aminobutyric acid (GABA) (Borbély et al., 2018). Moreover, extended periods of wakefulness in human studies are better associated with increased IEDs, particularly in generalized epilepsy syndromes compared to sleep duration (Stirling et al., 2023).

Recent fMRI studies have shown that sleep deprivation impairs thalamocortical connectivity, thus reducing the propensity of the brain to enter and stay in deeper stages of sleep (Nilsonne et al., 2017; Verweij et al., 2014). DTI studies in sleep-deprived epileptic subjects demonstrate microstructural alterations of thalamic and ARAS-related white matter tracts, further reinforcing the concept of a thalamocortical dysregulation as part of the increased seizure susceptibility following sleep loss (van den Munckhof et al., 2020). These findings suggest that homeostatic and circadian regulation of sleep is critical in maintaining the delicate balance of excitability within the epileptic brain. Table 1 provides an overview of sleep-epilepsy interactions and findings derived from various study models.

**Table 1:** Overview of sleep-epilepsy interactions and findings from various study models.

Study type	Key findings on sleep-epilepsy interaction	Techniques used	References
*In vitro*	Increased cortical excitability under sleep deprivation-like conditions Increased interictal discharges due to altered ion channels	Cellular imaging Electrophysiology	Lin et al. (2020)
*In vivo* (animal)	Increased seizure susceptibility with sleep deprivation Circadian modulation of seizure threshold	Electroencephalography (EEG) Genetic modulation	Purnell et al. (2021) and Borbély et al. (2018)
Human (sleep stages)	Non-rapid eye movement (NREM) sleep increases seizure activity Rapid eye movement (REM) sleep suppresses it Sleep deprivation elevates cortical excitability	EEG MEG Functional magnetic resonance imaging (fMRI)	Frauscher et al. (2015) and Ng and Pavlova (2013)
Human (microstructure)	Cyclic alternating patterns (CAP) phase A1 linked to increased interictal epileptiform discharges (IEDs) Spindle disruptions impact memory High-density IEDs reduce hippocampal spindles	EEG MRI Electrocorticography (ECoG) Diffusion tensor imaging (DTI)	Gelinas et al. (2016), Kramer et al. (2021), and Parrino et al. (2014)
Human (circadian)	Circadian genes modulate seizure timing, with specific peaks in epilepsy types Thalamic dysregulation in nocturnal seizures	EEG MEG DTI fMRI	Frauscher et al. (2015), Liu et al. (2022), and Re et al. (2020)

The interfaces between sleep and epilepsy highlighted here support a more detailed analysis of how sleep disruption in epilepsy may impair fundamental neurophysiological processes, most notably glymphatic function. The glymphatic system serves to clear metabolic waste from the brain, particularly during SWS and requires intact and stable sleep architecture to function optimally. Disrupted stages of sleep, arousals, and fragmented cycles, however, are features of epilepsy and may result in less-than-optimal glymphatic clearance, i.e., glymphopathy. The glymphopathy might further enhance neuroinflammation and increase the accumulation of neurotoxic metabolites, thus creating a pro-epileptogenic environment and enhancing seizure susceptibility. In turn, this is likely to have an impact on other sleep-related processes of waste clearance and neuroprotection besides Aβ, accelerating cognitive decline and neurodegeneration, particularly in the presence of comorbid CSVD. The intersection of sleep, glymphopathy, and CSVD in epilepsy thus outlines a pathophysiological framework whereby sleep-dependent mechanisms of clearance are related to the progressive nature of epilepsy and its respective comorbidities. This realization might indicate a potential therapeutic role of enhancement of glymphatic activity and sleep-focused interventions in improving outcomes in patients with epilepsy.

## 3 Glymphatic system, sleep, and epilepsy

It is important to note that not all epilepsy patients experience a reduction in SWS. The presence of IEDs alone, in the absence of clinical seizures or other sleep disorders, does not necessarily induce microarousals significant enough to prevent the initiation or maintenance of SWS. Therefore, while SWS disruption is frequently reported in epilepsy, its impact on brain clearance of toxic compounds is more likely attributed to molecular-level mechanisms, such as neuroinflammation and altered neurotransmission, rather than direct alterations in SWS itself.

The glymphatic system acts mainly during sleep and serves as the pathway for waste clearance. Its activity is paramount for neural homeostasis, which eliminates metabolic by-products and neurotoxic substances from the brain. Astrocytic end-feet and its channels, perivascular spaces, and cellular interactions depend on the expression of a water channel protein, such as aquaporin-4 (AQP4), which enables fluid exchange. Recent data have emerged that demonstrate glymphatic dysfunction or glymphopathy in a spectrum of neurological disorders, including epilepsy, wherein impaired clearance worsens neuroinflammation, oxidative stress, and seizure susceptibility (Iliff et al., 2012; Voumvourakis et al., 2023; Lee et al., 2022a; Lee et al., 2022b). Glymphopathy plays a crucial role in the underlying mechanisms and the development of epilepsy and may offer new therapeutic perspectives for improving seizure control and preserving cognition.

### 3.1 Glymphatic system: role of astrocytes and AQP4

The most active role of astrocytes in the glymphatic system involves the formation of perivascular channels, in which CSF enters the brain interstitium to promote the removal of metabolically active waste. AQP4 is a water channel protein expressed abundantly on astrocytic end-feet; it is critical to this pathway. AQP4 provides an efficient conduit for the movement of CSF along perivascular spaces, driving the convective flux necessary for the clearance of solutes, including Aβ and tau (Iliff et al., 2012). Altered *Aqp4* gene expression was one of the shared findings in epilepsy and more often in areas with intense seizure activities, suggesting that impaired water transport and glymphopathy might contribute to neuroinflammation and epileptogenesis (Bonosi et al., 2023).

For example, in animal models, genetic deletion of the *Aqp4* gene (i.e., *Aqp4* knockout) reduces glymphatic flow to bring forth the clinical manifestations of accumulated toxic metabolites and increased susceptibility to seizure activity (Gomolka et al., 2023; Mestre et al., 2018). In human research, recent advancements in neuroimaging such as fMRI and positron emission tomography (PET) have been able to show that patients with epilepsy often have impaired glymphatic clearance, as documented by reduced CSF flow in perivascular spaces and with increased retention of neurotoxic proteins (Goodman and Szaflarski, 2021). Thus, these findings support the hypothesis that astrocytic dysfunction together with altered expression of AQP4 can further exacerbate the neurotoxic environment during epilepsy, promoting neuronal hyperexcitability and seizure propagation.

### 3.2 Role of pericytes, microglia, and the blood–brain barrier

The pericytes are contractile cells along the walls of blood vessels and, in this respect, play an important role in cerebral blood flow and the integrity of the blood–brain barrier (BBB). They also support the glymphatic function by regulating CSF influx and waste clearance (Eltanahy et al., 2021). Pericytes are endothelial cells that wrap around the brain’s microvessels, playing an active role in the BBB structure and function. Such pericyte dysfunction in epilepsy is associated with increased permeability across the BBB, leading to the infiltration of inflammatory molecules and immune cells into the brain parenchyma, disrupting further glymphatic flow, and contributing to a seizure-prone environment (Yamanaka et al., 2021). Indeed, disruption of the BBB has been observed both in human and animal models of epilepsy, along with increased perivascular inflammation and microbleeds (Han et al., 2024).

Besides, the microglia in the glymphatic system, whose principal job is that of resident immune cells in the brain, regulate cellular debris clearance. In sleep, microglia assume a surveillance phenotype that monitors and clears damaged cells or molecules which may induce neuroinflammation. In epilepsy, microglia usually assume the active form releasing pro-inflammatory cytokines, leading to increased tissue damage and neurotoxicity (Hiragi et al., 2018). This may lead to further advancement of the pro-epileptogenic environment by impairing glymphatic efficiency and, hence accelerating cognitive decline (Zeng et al., 2022). Indeed, such findings would support that microglial activation, dysfunction of the BBB, and impaired pericyte function together contribute to glymphopathy in epilepsy. Table 2 provides an overview of key cellular and molecular players in glymphatic function and their roles in epilepsy.

**Table 2:** Key cellular and molecular players in glymphatic function and their roles in epilepsy.

Component	Role in glymphatic function	Dysregulation in epilepsy	References
Astrocytes	Form perivascular channels Facilitate cerebrospinal fluid (CSF) flow	Impairs waste clearance Promotes inflammation	Iliff et al. (2012) and Mestre et al. (2018)
Aquaporin-4 (AQP4)	Mediates water and fluid movement in and out of astrocytes Major channel for glymphatic flow	Altered expression linked to increased seizure susceptibility	Bonosi et al. (2023), Gomolka et al. (2023), Goodman and Szaflarski (2021), Iliff et al. (2012), and Mestre et al. (2018)
Pericytes	Regulate cerebral blood flow and blood–brain barrier (BBB) integrity	Dysfunction leads to BBB disruption Inflammation	Eltanahy et al. (2021) and Han et al. (2024)
Microglial	Clear debris and support an anti-inflammatory state in sleep	Chronic activation exacerbates neuroinflammation	Hiragi et al. (2018) and Zeng et al. (2022)

### 3.3 Glymphopathy in epilepsy: impact of neuroinflammation and oxidative stress toward seizure propagation and epileptogenesis

Epilepsy is generally characterized by a condition of chronic neuroinflammation, potentially caused by the impairment in glymphatic clearance of pro-inflammatory molecules. Normally, the flow of glymphatic would clear cytokines and other inflammatory molecules from the brain, thereby taking part in maintaining an anti-inflammatory environment. In epilepsy, glymphopathy might promote the local accumulation of such molecules on the one hand, hence enabling a pro-inflammatory environment to establish itself; this may again contribute to seizure activity itself (Sanz et al., 2024; Vezzani et al., 2011).

This inflammatory state is further fostered by the activation of microglia by releasing cytokines like IL-1β and tumor necrosis factor-alpha (TNF-α), which are noted to enhance neuronal excitability and promote seizure spread (Hiragi et al., 2018). *In vitro* studies have exposed that the pro-inflammatory cytokines may directly affect the ion channels of neurons and enhance the tendency toward spontaneous epileptiform discharges (Galic et al., 2012). Animal models with glymphopathy express higher levels of oxidative stress markers and more frequent spontaneous seizures, a fact that underlines the relationship between glymphopathy, neuroinflammation, and epilepsy *in vivo* (Peng et al., 2024). Moreover, recent studies also indicate that a multiprotein complex such as NOD-, LRR- and pyrin domain-containing protein 3 (NLRP3) inflammasome activity co-occurs with microglial activation and glymphopathy coordinates the IL-1β and IL-18 production in response to tissue damage, cellular stress, and infection also plays an important role in seizure susceptibility and epileptogenesis (Chen et al., 2024; He et al., 2021).

This becomes important because impaired clearance of neurotoxic substances, including Aβ and tau in epilepsy, is highly relevant to seizure propagation and epileptogenesis. The accumulation of Aβ, normally associated with neurodegenerative diseases, has recently been observed in epilepsy patients, in particular those suffering frequent seizures with cognitive decline (Romoli et al., 2021). Thus, glymphopathy in epilepsy may accelerate neurodegeneration, establishing a vicious cycle by which neuronal injury further promotes seizure activity. DTI studies in epilepsy patients reveal microstructural damage in white matter pathways associated with the hippocampus, a key site of seizure initiation and propagation (Lin et al., 2020). This may suggest that glymphopathy provides not only for the neurodegenerative development of the pathological process but also for seizure spread within the brain.

Moreover, genetic studies have shown that mutations in genes involved in astrocytic and glymphatic function, including AQP4 and a gene that encodes the excitatory amino acid transporter 2 (EAAT2) protein such as *SLC1A2*, may provide susceptibility to epilepsy via perturbation of glymphatic efficiency. EAAT2 is a membrane-bound protein that clears glutamate, an excitatory neurotransmitter, from the extracellular space at synapses in the brain (Alijanpour et al., 2023). Additionally, mice with *Aqp4* knockout displayed reduced glymphatic flow and enhanced susceptibility to seizures induced by kainic acid (Sun et al., 2024), thus demonstrating the molecular link between glymphopathy and the risk of developing epilepsy.

### 3.4 Glymphatic function, sleep architecture, and epilepsy

Active during SWS, the glymphatic system depends upon astrocytic AQP4 channels to promote bulk flow of CSF into the brain parenchyma, thereby enhancing metabolic waste removal (Lee et al., 2022a; Lee et al., 2022b; Voumvourakis et al., 2023). Sleep disruption compromises this vital function. Studies using both fMRI and PET imaging have shown that, in epilepsy patients, sleep often means reduced glymphatic activities and, therefore increased neurotoxic metabolite concentrations. For example, PET studies have shown reduced glymphatic clearance during SWS (Goodman and Szaflarski, 2021), a finding reproduced by EEG studies which showed fragmented SWS in them (Hablitz et al., 2019).

**Figure 2:**
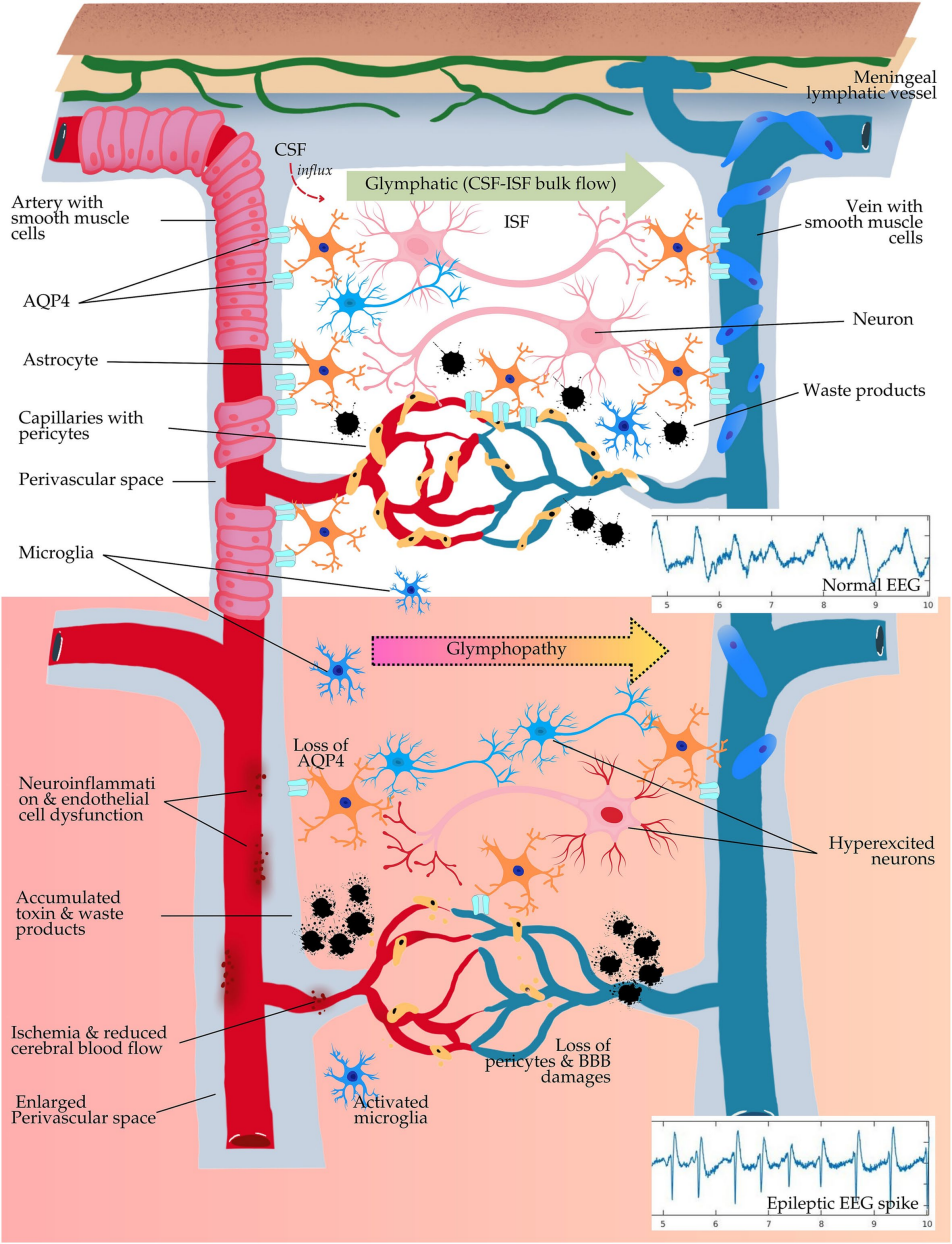
Schematic illustration of the glymphatic system and cellular interactions in epilepsy. This schematic illustrates the glymphatic system’s role in clearing brain waste and how dysfunction, termed “glymphopathy,” contributes to epilepsy. Normal state (top): cerebrospinal fluid (CSF) flows through aquaporin-4 (AQP4) channels in astrocytes, supporting waste clearance. The healthy blood–brain barrier (BBB) and neuro-glial-vascular function maintain stable neuronal activity, as shown in regular electroencephalography (EEG) spikes. Glymphopathy (middle to bottom): impaired glymphatic flow, loss of AQP4, and damaged BBB lead to toxin buildup, neuroinflammation, and vascular dysfunction. This disrupts neuronal environments, leading to hyperexcitability and epileptic EEG spikes. The accumulation of waste and vascular damage is implicated in epilepsy development, as glymphatic failure promotes conditions that trigger seizures.

This is further corroborated by disrupted sleep architecture, which, through reduced SWS and increased arousals, especially decreases the efficiency of glymphatic clearance. In animal models, sleep fragmentation and sleep deprivation reduce glymphatic flow by about 60%, leading to the accumulation of Aβ and tau, which are known contributors to cognitive impairment and epileptogenesis (Lee et al., 2022a; Lee et al., 2022b). Additionally, a recent study using diffusion tensor image analysis along the perivascular space (DTI-ALPS) index, i.e., the imaging-based mathematical method used as an indirect measurement of glymphatic activity, indicated that poor sleep disrupts glymphatic clearance, adding to memory decline. These findings provide important evidence that the quality and architecture of sleep affect cognitive health through underlying neural interactions and the interplay between the glymphatic system and the multimodal brain networks (Ma et al., 2024). In summary, such findings would support the idea that sleep-related dysfunction in glymphatic function might be a critical link between sleep disturbances and progressive neurological damage in epilepsy. Figure 2 illustrates the role of the glymphatic system and cellular interactions in epilepsy.

However, further research should focus on the precise molecular mechanisms linking glymphopathy, epileptogenesis, and cerebrovascular disease particularly CSVD, as well as exploring gene–environment interactions that may predispose individuals to glymphatic-related epilepsy and vascular pathology. Advanced neuroimaging techniques, such as DTI and PET-fMRI fusion, offer promising tools to assess glymphatic function and vascular integrity *in vivo*, aiding in the development of personalized therapies that target glymphatic restoration and reduce CSVD-associated risks.

## 4 CSVD in the context of epilepsy and sleep

CSVD is a neurovascular disorder affecting cerebral microcirculation, namely, arterioles, capillaries, and venules, leading to white matter lesions or white matter hyperintensities (WMHs), lacunar infarcts, and microbleeds seen on neuroimaging (Duering et al., 2023). CSVD is highly prevalent among older adults; thus, studies have estimated that up to 60% of individuals over 60 exhibit some form of CSVD-related pathology (Wardlaw et al., 2019). Alarmingly, CSVD is often occult or silent, as the majority of individuals do not show any symptoms, for instance, a recent cross-sectional study found that 30% of asymptomatic individuals aged 25 to 60 years showed evidence of CSVD in neuroimaging (Ghazali et al., 2022). There is increasing evidence that CSVD contributes to the pathophysiology not only of cognitive impairment and neurodegenerative diseases but also of epilepsy and sleep disturbances and vice versa. CSVD, epilepsy, and sleep disturbances interact in a complex interplay of neurovascular, neuroinflammatory, and glymphopathy, leading to a worsening of the clinical outcome for each condition.

### 4.1 Pathophysiology of CSVD in epilepsy

CSVD exerts neurovascular dysfunction through chronic hypoperfusion, breakdown of the BBB, and thrombo-inflammation. As discussed, in the context of epilepsy, these vascular abnormalities promote cortical excitability and disrupt neural homeostasis, further deteriorating seizure risk. Additionally, impaired oxygen and nutrient delivery due to chronic hypoperfusion in CSVD results in metabolic stress and impairment of synaptic function, which therefore may increase the susceptibility in the brain to seizures (Turon et al., 2021).

As stated, the integrity of BBB is compromised in CSVD, allowing neurotoxic proteins, inflammatory cytokines, and immune cells to penetrate the brain parenchyma. Such an inflammatory milieu is associated with the release of pro-epileptogenic factors, including IL-1β and TNF-α, that enhance neuronal excitability and lower seizure thresholds (Villasana-Salazar and Vezzani, 2023). Animal models have shown that the induction of BBB disruption is sufficient to cause spontaneous epileptiform activity, further implicating vascular compromise in epileptogenesis (Greene et al., 2022). Apart from that, several human brain imaging studies using DTI have revealed microstructural changes in white matter tracts including ARAS and AAS-related pathways are associated with CSVD in epilepsy patients, suggesting a neurovascular contribution to both epileptic activity and cognitive decline (Leyden et al., 2015).

CSVD is further characterized by enhanced oxidative stress, microthrombus accumulation and endothelial dysfunction, factors well known to exacerbate neuro-thrombo-inflammation and neuronal damage (Lee et al., 2021). Such changes provide a pro-epileptogenic setting, as *in vivo* studies demonstrated heightened seizure susceptibility in mice with induced endothelial dysfunction (Huang et al., 2022). Altogether, data provided suggest that neurovascular disturbance due to CSVD compromises brain function but also promotes seizure activity in susceptible individuals, such as people with epilepsy.

### 4.2 CSVD and glymphopathy

The effect of CSVD further extends to the glymphatic system or vice versa; pathology in small vessels disrupts perivascular fluid dynamics, which plays a critical role in waste clearance. For the glymphatic system, an intact vascular network is required, along with sufficient arterial pulsatility, to drive onward the movement of CSF through the perivascular spaces. In the setting of CSVD, reduced vascular compliance and flow impede this process of clearance, leading to the accumulation of metabolic waste and neurotoxic substances such as Aβ and tau (Csiszar et al., 2024). This accumulation is attributed to enhanced neuroinflammation that can further worsen epilepsy and help contribute toward a decline in cognition among patients.

Besides, pericytes play an indispensable role in maintaining the integrity of the BBB and in the regulation of the capillary blood flow. Thus, their presence means a lot in terms of efficient glymphatic functioning. Pericyte loss or dysfunction commonly occurs in CSVD and leads to disturbed waste clearance with increased inflammatory signaling (Eltanahy et al., 2021). Multiple studies indeed have demonstrated that the deletion of pericytes leads to impaired glymphatic flow (Eltanahy et al., 2021) and increased susceptibility to seizures (Yamanaka et al., 2021), hence linking vascular dysfunction of CSVD with glymphatic impairment in epilepsy. Pre-clinical (small animal) and clinical (human) studies using advanced MRI techniques have shown reduced CSF clearance in coupled with CSVD, indicating a glymphopathy contributes to a pro-epileptogenic environment through neuroinflammation and metabolic stress (Majercikova et al., 2023; Tumani et al., 2015; Turon et al., 2021; Wang et al., 2024).

Furthermore, activated microglia are considered important regulators of the glymphatic flow because of their capability of cellular debris removal in response to vascular pathology and accumulation of waste. In CSVD, however, this turns into chronic activation that leads to excessive release of inflammatory cytokines damaging neuronal networks and hindering further glymphatic clearance. It has also been suggested that chronic microglial activation may contribute to both epileptic seizures and disturbed sleep, with a feed-forward mechanism whereby each glymphopathy and neuroinflammation self-perpetuates to further exacerbate epilepsy and cognitive impairment (Devinsky et al., 2013).

### 4.3 Implications of CSVD on epileptic outcomes

As aforementioned, these factors point toward a convergence of CSVD, glymphopathy, and epilepsy, a factor of great implications for clinical outcomes, especially cognitive and neurological decline. In epilepsy comorbid with CSVD, there is a further risk of accelerated cognitive impairment; white matter lesions of CSVD and glymphopathy further exacerbate neuroinflammatory processes and neuronal damage. A recent systematic review and meta-analysis (Doerrfuss et al., 2023) and studies with MRI in patients with epilepsy and CSVD (Lin et al., 2020), indicated that there is widespread white matter abnormality, often associated with disturbances in executive function, memory, and attention.

Emerging research indicates a link between CSVD and an increased risk of epilepsy in older adults, with studies showing that elderly patients with CSVD and epileptic seizures have a significantly higher prevalence of juxtacortical small lesions (80.5%) compared to those without seizures (22.0%), identifying these lesions as strong independent predictors of seizures (Turon et al., 2021).

Indeed, apart from cognitive decline, sleep disturbances are more prevalent in epilepsy patients with vascular diseases such as CSVD (Gutter et al., 2019). Both conditions presented disrupted sleep architecture, as manifested by decreased SWS and increased wake after sleep onset. This further aggravates sleep quality by reducing the efficiency of glymphatic clearance and enhancing the accumulation of neurotoxic waste, therefore increasing seizure vulnerability. In contrast, epilepsy patients with neurodegenerative diseases such as CSVD and Alzheimer’s revealed decreased clearance of Aβ during sleep, suggesting that glymphopathy contributes to seizure susceptibility and long-term neurodegeneration (Szabo et al., 2022).

Furthermore, epilepsy has been associated with increased cardiovascular morbidity and mortality. Moreover, in the case of CSVD, the efficacy of antiepileptic drugs (AEDs) might be impeded because of disrupted BBB which may cause variable drug delivery and less than optimum seizure control (Dorszewska et al., 2016; Katsiki et al., 2014). Increased drug efflux is associated with BBB permeability in CSVD since the inflammatory processes upregulate the efflux transporters across the BBB, thereby possibly reducing the efficacy of AEDs. This all constitutes a challenge for clinicians who manage epilepsy in patients with comorbid CSVD since the conventional AED regimen might be inadequate for seizure control in such a population (Dorszewska et al., 2016; Katsiki et al., 2014).

All in all, understanding the interplay between the glymphatic system, sleep, CSVD, and epilepsy is crucial for delineating the underlying pathophysiological mechanisms that contribute to seizure susceptibility, neuroinflammation, and cognitive decline. By examining how glymphopathy, vascular integrity and sleep architecture interact, researchers can better identify the molecular and cellular pathways that exacerbate epileptogenesis and neurological deterioration in patients with these comorbid conditions. This integrated approach not only advances our understanding of the complex relationship between these systems but also opens new avenues for therapeutic interventions aimed at improving glymphatic clearance, supporting vascular health, and optimizing sleep quality, ultimately offering hope for more effective, personalized treatments in epilepsy management.

## 5 Interplay between sleep, glymphatic system, CSVD, and epilepsy: a mechanistic model

**Figure 3:**
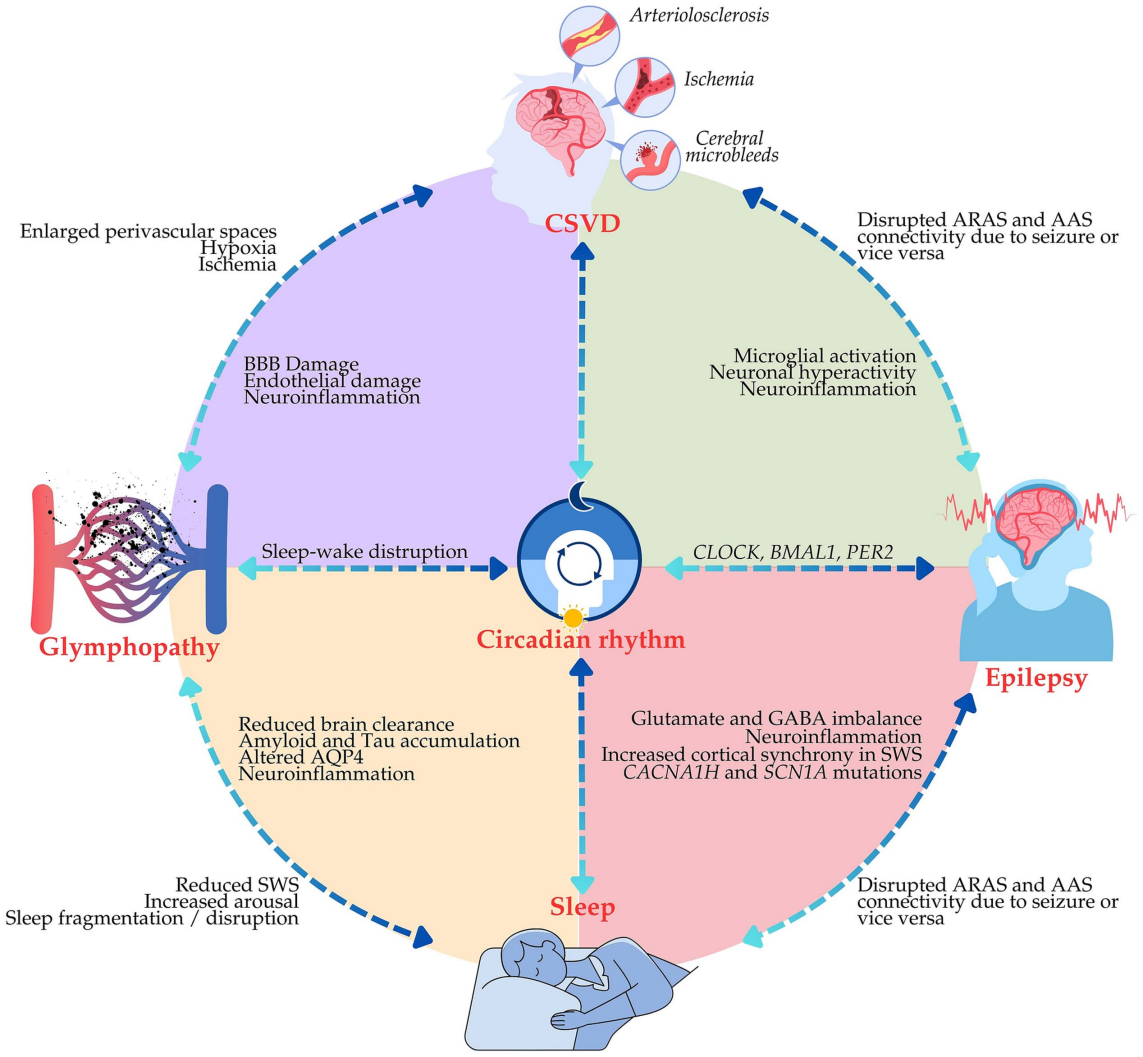
The proposed mechanistic model of interactions between sleep, the glymphatic system, cerebral small vessel disease (CSVD), and epilepsy. The illustrative diagram shows a feedback loop in which sleep disruptions impair glymphatic clearance, leading to neurotoxic accumulation and neuroinflammation, which exacerbate CSVD and contribute to epilepsy.

Emerging evidence highlights the intricate interplay between sleep quality, glymphatic function, CSVD, and epilepsy, suggesting a shared mechanistic platform driving disease progression and neurodegeneration. Disruptions in one system can cascade into dysfunction in others, exacerbating epileptogenesis and cognitive decline. Here, we present a simplified mechanistic model (Figure 3) illustrating the reciprocal influences among these systems, emphasizing their collective impact on neuroplasticity, neuroinflammation, and seizure susceptibility.

### 5.1 Sleep-glymphatic function interactions and their role in epilepsy

SWS plays a crucial role in glymphatic clearance, as AQP4 astrocytic channels facilitate CSF flow through perivascular spaces to remove neurotoxic waste (Nedergaard and Goldman, 2020; Voumvourakis et al., 2023). Sleep disturbances in epilepsy, such as fragmented sleep and reduced SWS, may lead to glymphopathy (Garg et al., 2022; Nobili et al., 2022), leading to the accumulation of neurotoxic metabolites that fuel neuroinflammation and neuronal hyperexcitability, the key contributors to seizure generation.

The presence of CSVD exacerbates these processes by restricting cerebral blood flow and vascular compliance, further impairing glymphatic clearance and accelerating toxic protein accumulation. The resulting buildup of neurotoxic waste drives neuroinflammation and establishes a pro-epileptogenic milieu-as evidenced by enhanced seizure vulnerability in animal models with impaired glymphatic function (Cai et al., 2024; Sun et al., 2024). Clinical studies also demonstrate that patients with co-occurring epilepsy and CSVD exhibit more severe glymphopathy and heightened seizure susceptibility, reinforcing the link between vascular dysfunction and epileptogenesis (Doerrfuss et al., 2023; Lin et al., 2020).

### 5.2 Neuroplasticity, neuroinflammation, and epileptogenesis

Sleep disruptions, glymphatic inefficiency, and CSVD collectively impair synaptic downscaling and memory consolidation, fostering maladaptive neuroplasticity that increases seizure risk. IEDs disrupt synaptic homeostasis, driving hyperconnectivity in epileptogenic circuits. Additionally, failure to clear excess neurotransmitters and inflammatory proteins further compromises neural connectivity, leading to long-term structural changes that lower seizure thresholds (Horvath et al., 2020; Moraes et al., 2021).

Chronic neuroinflammation, driven by glymphatic dysfunction and BBB breakdown, triggers a vicious cycle where inflammatory mediators (e.g., IL-1β, TNF-α) enhance neuronal excitability, contributing to seizure propagation (Devinsky et al., 2013). Experimental models confirm that impaired glymphatic function induces spontaneous seizures and exacerbates neuroinflammatory damage, supporting its role in epilepsy progression (Peng et al., 2024).

### 5.3 Proposed mechanistic model: a vicious cycle of neurovascular and glymphopathy in epilepsy

In the proposed mechanistic model, disrupted sleep triggers a downward spiral of glymphopathy and increased CSVD pathology, each contributing separately to epileptogenesis and cognitive decline. Poor quality sleep, especially reduced SWS, reduces glymphatic efficiency and leads to the accumulation of neurotoxic substances that further provoke neuroinflammation. Neuroinflammation disrupts neurovascular function and further worsens the pathology associated with CSVD. The resultant neurovascular dysfunction further reduces glymphatic clearance and enhances seizure susceptibility, thereby constituting a self-sustaining cycle of accelerating disease in epilepsy. Figure 3 illustrates the putative mechanistic model of interactions between sleep, glymphatic system, CSVD, and epilepsy.

Overall, this mechanistic model serves as the backbone for future studies that focus on developing holistic, personalized approaches to epilepsy management. These pathways need further validation in clinical populations, and such future studies should also explore advanced neuroimaging like PET-MRI and DTI assessing the *in vivo* efficiency of glymphatic and vascular systems. This may allow clinicians to target the root mechanisms of epilepsy progression per se, thereby improving outcomes for patients so afflicted by these interrelated disorders.

## 6 Clinical implications and therapeutic opportunities

Indeed, this is a complex interplay that sleep, glymphatic function, CSVD, and epilepsy enter into, justifying the need for an integrated approach in diagnosis and therapy. Interventions targeting sleep architecture and neurovascular health might have added value in seizure control but, equally important, in cognitive and neurological decline associated with epilepsy and its comorbid conditions. Advanced diagnostic and personalized treatment options thus represent the most promising avenues of optimisation in patients.

### 6.1 Diagnostic and monitoring innovations

Management thus requires comprehensive assessment methodologies due to the complex interplay among sleep disruption, glymphopathy, and CSVD in epilepsy. Sleep studies, together with molecular and structural imaging of glymphatic function and markers of CSVD, may provide important insights into individual patients’ pathophysiology and thus be guided toward targeted interventions.

In sleep studies, the use of EEG, polysomnography (i.e., a diagnostic test that tracks and records how multiple body systems work during sleep) and actigraphy (i.e., non-invasive method using an actimetry sensor to monitor human rest/activity cycles); either in combination or stand-alone, represent useful investigations that provide information on sleep architecture and details about specific disturbances in SWS and REM sleep. Several studies have documented that sleep fragmentation and reduction of SWS are frequent in epilepsy patients, particularly in those with CSVD, which per se is associated with increased seizure frequency and greater cognitive impairment (Häusler et al., 2020; Sivathamboo et al., 2020). This may potentially enable the selection of those patients with epilepsy who are at increased risk for glymphopathy and permit appropriate sleep-based therapies targeted to the individual.

Moreover, in terms of glymphatic function imaging, imaging modality development like DTI-ALPS and dynamic contrast-enhanced MRI (DCE-MRI) allows for *in vivo* imaging of glymphatic function. While DTI-ALPS can detect structural changes in white matter tracts associated with glymphopathy (Ma et al., 2024), DCE-MRI can give insights into the flow of CSF and the efficiency of waste clearance (Benveniste et al., 2021). Indeed, multiple studies have provided evidence of impaired CSF flow and reduced efficiency in the removal of waste products (Iliff et al., 2012; Nedergaard and Goldman, 2020). PET has also been used to study Aβ removal in sleep-deprived epilepsy patients, showing glymphopathy and increased neurotoxic protein burden (Benveniste et al., 2021). Such studies may be further combined with glymphatic imaging to understand better how sleep disruption affects waste clearance in epilepsy.

Furthermore, neuroimaging tools such as MRI and fMRI can detect and quantify the presence of white matter hyperintensities, lacunar infarcts, and microbleeds as markers of CSVD. These are common in the elderly population and epilepsy patients, and their presence is related to worse cognitive outcomes and increased seizure burden (Turon et al., 2021). BBB permeability, as tested by gadolinium-enhanced MRI, may also reveal early signs of BBB dysfunction associated with CSVD and therefore be useful for predicting epilepsy progression and informing intervention strategies that protect vascular health (Andrijauskis et al., 2023).

### 6.2 Targeted therapeutic strategies

Therapies that target the unique interactions among sleep, glymphatic function, CSVD, and epilepsy may offer great opportunities for improving outcomes in patients. Recent research suggests that treatment focused on improving sleep stability, modulating glymphatic clearance, and supporting neurovascular health may reduce seizure susceptibility, mitigate cognitive decline, and improve the overall quality of life for epilepsy patients.

#### 6.2.1 Sleep-based glymphatic enhancement

Promotion of SWS pharmacologically and/or behaviourally might improve glymphatic clearance, lower accrual of neurotoxicants, and thus reduce seizure risk. SWS plays an important role in the efficacy of glymphatic functioning, whereby AQP4 channels on astrocytes are more functionally active during this stage of sleep, promoting the CSF flow via perivascular spaces. Accordingly, medications such as gaboxadol, a GABA agonist known to increase SWS, have shown some promise in enhancing glymphatic flow in animal models and may have potential applications in the management of epilepsy (McDonald et al., 2007; Walsh et al., 2008).

Aside from the promotion of SWS, chronotherapy has also been shown to be effective. Timing of the therapeutic interventions to fall concomitantly with patients’ circadian rhythms might provide some scope both for improving sleep quality and reducing seizure frequency (Cardinali et al., 2021). It is well recognized that circadian disruption increases seizure susceptibility, and chronotherapy aims at entraining sleep–wake cycles with the view to decrease that susceptibility. A study by Khan A. A. et al. (2018) further demonstrated that seizure timing is likely to be influenced by the circadian rhythm, with nocturnal seizures being more prevalent in epilepsy patients who have disrupted sleep cycles. Chronotherapy might help support the glymphatic clearance during SWS and thus may have dual benefits both in sleep and seizure management. Moreover, a recent *in vivo* study demonstrated that the uses of a non-psychoactive phytocannabinoid such as cannabidiol (CBD) had the potential to restore the excitability of hippocampal neurons and protect against cellular damage in epilepsy models, hence it may act as an anti-seizure medication to normalize brain network function in such models (Khan S. et al., 2018).

#### 6.2.2 Anti-CSVD interventions

Improvement in vascular compliance and protection of BBB integrity might avoid glymphopathy in CSVD-related treatments, thereby reducing neuroinflammation and seizure susceptibility. Thus, many agents used in clinical practice, including but not limited to statins and angiotensin receptor blockers, have been shown to protect endothelial function and therefore may mitigate the rise in BBB permeability, thus encouraging vascular health in epilepsy with coexistent CSVD (Doege et al., 2022; Trivedi et al., 2018). Moreover, the elastin model is crucial for understanding vascular elasticity and remodeling, while minoxidil, through its vasodilatory effects, may influence elastin synthesis and arterial stiffness, offering potential therapeutic insights for cerebrovascular diseases. For example, a recent *in vivo* study reported that the administration of minoxidil in elastin (Eln+/−) mice can improve vascular compliance, restore cerebral blood flow and enhance perivascular flow (Knutsen et al., 2018), which may, in turn, promote glymphatic function and prevent the buildup of pro-epileptogenic factors.

The pericyte and endothelial cells have been strongly implicated in the support of neurovascular health and glymphatic efficiency. Targeting pericyte function with therapies that enhance cellular resilience, and repair may serve to restore vascular integrity and reduce glymphopathy in CSVD. Furthermore, various experimental treatments aimed at increasing the survival of endothelial cells and reducing oxidative stress might further alleviate disruption to the BBB caused by CSVD and hence reduce seizure risk in epilepsy patients with compromised vascular health.

#### 6.2.3 Anti-inflammatory and neuroprotective approaches

Modulation of microglial activity might provide one complementary target for treatment associated with neuroinflammation in epilepsy. Indeed, chronic activation of microglia, generally exacerbated by glymphopathy, predisposes an individual to neuroinflammation, thus increasing seizure susceptibility. Anti-inflammatory agents or immunomodulatory therapies directed against microglial activation could reduce the release of pro-inflammatory cytokines, such as IL-1β and TNF-α, known to enhance neuronal excitability (Krause and Müller, 2010; Shi et al., 2013). It is through animal model studies that anti-inflammatory treatments suppress seizure frequency and protect cognitive function to point out the potential outcome in epilepsy patients with glymphatic and neurovascular co-morbidities (Peng et al., 2020; Peng et al., 2024).

Moreover, AQP4 modulation by targeting the AQP4 channels in astrocyte end-feet is another therapeutic approach that may support glymphatic clearance. Such pharmacological or gene-therapy-based modulation of AQP4 expression could enhance CSF flow via the perivascular spaces and diminish the buildup of neurotoxic products (Berliner et al., 2023), thus lowering seizure vulnerability. Consistent with the role of AQP4 in modulating seizure susceptibility *in vivo*, *Aqp4* gene deletion reduces glymphatic clearance and enhances seizure susceptibility in AQP4 knockout mice (Bonosi et al., 2023).

## 7 Current limitations and future research directions

Although knowledge is fast emerging on the interaction of sleep with epilepsy, and with the glymphatic system and CSVD, limitations in the current state of research and methodologies impede a comprehensive solution from being achieved. Identifying such limitations will help focus future research efforts on the gaps, advancement of diagnostic tools, and development of novel treatments. Here we outline key limitations in the study of these interactions and, where possible, further avenues of research.

### 7.1 Methodological challenges in assessing glymphatic and sleep function

The main challenge, so far, remains the direct assessment of the function of human subjects. Most of the investigations regarding glymphatic are indirectly made through the techniques of DTI-ALPS and contrast-enhanced MRI, which are not able to reveal real-time dynamics of fluid flow through the glymphatic system. The glymphatic system is most active during SWS, however, capturing these dynamics in real-time in humans is complex and heavily dependent on technological advancement (Nedergaard and Goldman, 2020). Besides, animal studies have potential limitations in replicating human physiology of the brain (Homberg et al., 2021), so translation from *in vivo* models to epilepsy and CSVD clinical practice remains rather challenging.

As for future recommendations, in this case, non-invasive imaging will be required to develop technologies that will enable the capture of real-time CSF flow, for example, advanced MRI modalities, which could measure glymphatic function during natural sleep. This may involve the use of wearable devices that can monitor sleep from home and accurately track stages of sleep. This could enhance the validity of sleep data and provide a better understanding of the different stages of glymphatic activity associated with disrupted patterns of sleep in epilepsy, such as SWS.

### 7.2 Limited understanding of glymphatic and neurovascular interactions

The relationship between glymphatic function and neurovascular health, in particular CSVD, is still under active investigation. Though some studies suggest that vascular compliance and impaired integrity of the BBB in CSVD disrupt the glymphatic flow, leading to neurotoxic accumulation, few studies link these mechanisms directly to epilepsy (Lee et al., 2022a; Lee et al., 2022b; Sun et al., 2024; Voumvourakis et al., 2023). Research up until now has focused most attentively on animal models of CSVD, with findings from animal models yet to be validated in human studies. This, in turn, limits our knowledge about how vascular changes influence glymphatic function and subsequent epileptogenesis.

It is recommended that future longitudinal studies on the ability of multimodality imaging in human populations to demonstrate vascular changes associated with CSVD are needed to unravel the underlying glymphatic function contribution to epilepsy. A combination of state-of-the-art MRI and PET imaging with vascular biomarkers would provide insight into how neurovascular health affects glymphatic clearance and seizure risk, especially in older adults and individuals with comorbid conditions.

### 7.3 Incomplete understanding of molecular pathways

Current studies also lack the specific molecular mechanisms that explain the linkage of sleep, glymphopathy, CSVD, and epilepsy. Moreover, though complex neuroinflammation and disrupted sleep-dependent plasticity have been advocated to contribute to epileptogenesis, there is a general shortage of knowledge regarding genetic and epigenetic factors that would make some people more predisposed to glymphatic-related epilepsy and CSVD. Furthermore, very limited data are available on how different genetic polymorphisms, i.e., single nucleotide polymorphism (SNPs) in genes modulating AQP4 expression, inflammatory cytokines, and circadian rhythm, will affect the interactions among those systems.

Future studies should be directed, with emphasis on molecular and genetic investigations to identify biomarkers and therapeutic targets in the sleep-glymphatic-CSVD-epilepsy axis. Genetic investigations into AQP4 SNPs and their influence on glymphatic clearance may explain individual variability in susceptibility to epilepsy and CSVD. Omics technologies, such as proteomics and metabolomics, may unravel the detailed signaling pathways and molecules participating in the interplay between sleep, neurovascular health, and epileptogenesis.

### 7.4 Limitations in biomarker development for early detection

Early diagnosis and timely intervention are limited by the fact that no validated biomarkers reflect the interrelated mechanisms of glymphopathy, sleep disturbances, CSVD, and epilepsy. No current imaging markers point to glymphopathy or sleep disturbances specifically, while molecular markers such as inflammatory cytokines are not yet clinically validated. Biomarkers capable of identifying and monitoring these interactions reliably are of utmost importance for early diagnosis, patient stratification, and treatment optimisation.

Hence, validation of new biomarkers that specifically link glymphopathy, neuroinflammation, and seizure susceptibility should be the direction of the studies. New blood, CSF, or imaging biomarkers reflecting glymphopathy and neurovascular health would be possible with advanced “omics” approaches such as metabolomics, proteomics, and inflammatory profiling. Biomarker validity testing in the course of epilepsy and CSVD would necessitate longitudinal studies for early diagnosis.

### 7.5 Challenges in developing personalized treatment approaches

Although evidence exists that sleep disruption, glymphopathy, and neurovascular health differ among individuals, very few current epilepsy treatments consider this individual variation (InformedHealth.org., 2023). Most current therapeutic interventions inadequately take into consideration sleep architecture or how the comorbidity of conditions such as CSVD impinges on glymphatic function. This lack of personalisation does have a risk of limiting the efficacy of treatments in such complex, multi-factorial epilepsy presentations.

Future clinical trials may study precision medicine strategies that provide personalized therapies, matched according to the individual’s sleep profile, glymphatic function, and neurovascular health. Other interventions are the modulation of sleep through a pharmacological intervention that targets SWS or behavioral interventions aimed at optimizing sleep architecture, which may enhance the outcomes of glymphatic clearance and seizure control. Vascular treatments such as statins, minoxidil, and angiotensin receptor blockers could be administered in a personalized manner among subjects with CSVD and epilepsy.

Moreover, given the complex bidirectional interaction between disrupted sleep and epilepsy, clinical trials targeting therapies that will improve sleep stability and SWS may hold the greatest promise of improvement in glymphatic function and seizure outcome. Novel sleep architecture therapies, such as gaboxadol to enhance SWS, or chronotherapies designed to optimize circadian rhythm, might offer additional benefits to individuals with co-occurring glymphopathy and CSVD associated with epilepsy. Neuroimaging and biomarker data may be beneficial to further delineate which patients are most likely to benefit from these approaches.

Future clinical trials may focus on combined interventions, including sleep-based glymphatic enhancement, anti-inflammatory therapies, and vascular protective agents, targeting the multifactorial needs of epilepsy patients with comorbid CSVD. Precision medicine models incorporating genetic, sleep, and neurovascular data may ultimately allow clinicians, in the future, to tailor treatment protocols to the individual, optimizing therapeutic responses and reducing disease burden.

Implementing these strategies in clinical practice presents challenges, including resource limitations and patient adherence. Advanced neuroimaging tools required for glymphatic assessment are not widely available, and patient reluctance to adopt sleep-focused interventions may hinder adherence. Addressing these barriers will require targeted education programs for clinicians and patients, as well as the integration of sleep assessment tools into routine epilepsy care.

### 7.6 Limitations of this review

This review therefore provides an overall outlook on the interaction between sleep and glymphopathy, CSVD, and epilepsy. This review is overtly limited by the breadth of available data and methodological constraints. Because large-scale longitudinal studies that explicitly test interactions among these systems are lacking, indirect evidence in some conclusions or findings from animal models must be used, incomplete to characterize fully the complexity of such relationships in human populations. Also, the current review predominantly relates to epilepsy and CSVD; further neurological comorbidities that might interfere with sleep and glymphatic function were not considered in this respect, including other neurodegenerative diseases such as Alzheimer’s disease and traumatic brain injury.

Such limitations may be overcome in future reviews that will be able to take into consideration the emerging data from clinical trials and human studies related to the glymphatic system, sleep interventions, and CSVD. One could also envision an expanded scope that elucidates how other neurodegenerative and neurological conditions interact with epilepsy, glymphopathy, and sleep.

## 8 Conclusion

Such an integrated understanding of the interplay between sleep, glymphatic function, CSVD, and epilepsy will be important for further advancing epilepsy management. It also becomes clear that disturbed sleep impairs glymphatic clearance, that CSVD promotes neuroinflammation, and that glymphopathy results in neurotoxic accumulation-each-enhancing seizure susceptibility and cognitive decline. The recognition of these links underlines the need for broad diagnostic and therapeutic approaches targeting the underlying neurovascular and glymphatic pathways in epileptogenesis. This should be the focus of future studies: advanced imaging techniques and biomarkers that allow the assessment of neuroinflammation, integrity of the blood–brain barrier, and glymphatic function enable the possibility of earlier detection and personalized monitoring of disease progression. Focused interventions-targeting therapies, including sleep-based therapies to enhance glymphatic clearance and treatments aimed at supporting vascular health-may improve seizure control, reduce cognitive decline and improve quality of life in epilepsy patients, particularly those comorbid for CSVD. Hence, this holistic and precision-oriented approach can revolutionize epilepsy care beyond seizure management to an all-encompassing, interrelating neurobiological basis for disease progression. Such a framework bridges advances in sleep science with neurovascular research in the treatment of epilepsy, thus affording new avenues for more effective and personalized patient care.
